# Glutamate facilitates root colonization by plant growth-promoting rhizobacteria *Bacillus subtilis* in tomato seedlings

**DOI:** 10.1128/spectrum.03181-25

**Published:** 2026-03-13

**Authors:** Rachel Warthen, Avaniek Cabales, Anna Wockenfuss, Charanpreet Kaur, Aditya M. Kunjapur, Harsh Bais

**Affiliations:** 1 Department of Plant and Soil Sciences, University of Delaware 536619, Newark, Delaware, USA; 2 AP Biopharma, University of Delaware 5972, Newark, Delaware, USA; 3 Department of Chemical and Biomolecular Engineering, University of Delaware 371258, Newark, Delaware, USA; Connecticut Agricultural Experiment Station, New Haven, Connecticut, USA

**Keywords:** PGPR, *Bacillus subtilis*, tomato, roots, mature region, surfactin, glutamate

## Abstract

The influence of microbially and plant-synthesized compounds on colonization of plant growth-promoting rhizobacteria (PGPR) on various regions of the plant root is underexplored. Here, we examine the influence of surfactin and glutamate on the level and specificity of colonization of *Bacillus subtilis* along different regions of tomato root by adding exogenous compounds and testing mutated bacterial strains. First, *B. subtilis* PY79 was modified to express a full-length phosphopantetheinyl transferase (*sfp*) native to other surfactin-producing *B. subtilis* strains, and surfactin biosynthesis was observed under static incubation at 25°C in plant culturing media. Microscopy was performed using PY79 sfp^−^ (surfactin null), PY79 sfp^+^ (surfactin overproducing), and the wild isolate *B. subtilis* UD1022 to map the colonization of each strain along the entire root of young tomato plants incubated at 25°C. In addition, experiments involving supplementation of surfactin or glutamate were also performed to evaluate the root colonization of sfp^+^ and sfp^−^ strains. Root mapping of a tomato seedling shows that *B. subtilis* prefers to colonize near the mature region of the root and that colonization patterns vary based on exogenous metabolite concentration. Inclusion of glutamate in the media or through transient priming of the plant prior to bacterial inoculation strongly promoted root colonization by *B. subtilis* strains (both sfp^+^ and sfp^−^). In addition, the data shows that the lab strains were less efficient in colonization compared to the wild-type *B. subtilis* strain. Interestingly, in the presence of glutamate, microbes lost their preference for colonization at the mature region, instead colonizing along the entire root. Overall, our work reveals a preference for colonization of these *B. subtilis* strains to the mature region of tomato in the absence of glutamate supplementation and demonstrates a smaller than anticipated role of biosynthesized or supplemented surfactin on root colonization, at least in a hydroponic culturing format.

**IMPORTANCE:**

Plants are associated with large communities of microbes across the rhizosphere. However, comparatively little is known about the drivers underpinning the diversity of microbes, especially regarding how they are recruited by plants. Growing evidence indicates that the rhizospheric microbiome supports plant growth in response to biotic and abiotic stresses. Of late, the usage of a synthetic community of plant growth-promoting rhizobacteria (PGPR), especially *Bacillus subtilis,* has been recognized for its role as a potential biofertilizer and bio-fungicide agent. The role of PGPR-derived metabolites has been debated as a driver for enhanced root colonization. However, the knowledge pertaining to where and how PGPR colonize on the root surface is currently unknown. Therefore, it is prudent to elucidate the role of bacterially derived compounds and other carbon sources in the rhizosphere that drive root colonization.

## INTRODUCTION

Plant growth-promoting rhizobacteria (PGPR) are known to induce both growth promotion and disease suppression in plants against classical bacterial and fungal pathogens. Among PGPR, several *Bacillus* spp. are used as biocontrol for various diseases that impact crops (1, 2). The interaction of *B. subtilis* with plants is driving novel areas of research that seek to understand how benign microbes interact beneficially with plants. The impact of fundamental research on *B. subtilis*-plant interactions has led to the commercialization of *B. subtilis* inoculums for sustainable agricultural practices (3). The difference in fitness and biofilm production between lab and wild strains of *B. subtilis* has been established, and the success of commercialized strains of *Bacillus* is often consequently linked to its ability to form root-associated biofilms (4, 5). Wild strains of *B. subtilis* often show poor ability to colonize both biotic and abiotic surfaces, suggesting a lack of key microbial components for colonization (6). It is argued that nutrient-rich media can facilitate the ability of *B. subtilis* 168-derived strains to form effective biofilms (7). On the contrary, wild strains of *B. subtilis* show colonization and biofilm formation on both abiotic and biotic surfaces.

Non-ribosomally synthesized peptides (NRPs) produced by *Bacillus* sp. exhibit antimicrobial properties, but their effect on biofilm formation and root colonization is less well understood (8). NRPs such as surfactin, iturin, and fengycin/plipastatin can disrupt cell membranes and cause cell death (8, 9). Among bacterially derived NRPs, surfactin is a cyclic lipopeptide, known to be important in its role for antimicrobial properties (9). Surfactin production in *B. subtilis* strains is regulated based on its interaction with the environment (10). The biosynthesis of surfactin in a few strains of *Bacillus* spp. shows increased levels of surfactin against a range of plant and soil-associated bacterial and fungal pathogens (11). The regulation of surfactin synthesis is well studied. Studies show that the surfactin biosynthetic mutants are equally capable of colonizing plants and abiotic surfaces (12). Fluctuations in surfactin production by *Bacillus* spp. and its impact on colonization of plant roots remain unclear. In addition, the role of surfactin in promoting biofilm formation by *Bacillus* sp. on both biotic and abiotic surfaces is known (13).

The ability of *Bacillus* spp. to colonize plant roots is dependent on *Bacillus* spp.’s ability to form microcolonies and establish biofilms (14). In addition to forming root-associated biofilms, several *B. subtilis* strains are also shown to form a floating biofilm called pellicles with distinct macroscopic architecture (15). It is shown that various plant-derived molecules activate a biofilm operon in *Bacillus* spp. that plays an important role in root colonization and pellicle formation (11, 16). Root-secreted malic acid from plants under stress recruits *Bacillus* spp. to form biofilms on plant roots (17). Other plant surface metabolites, including plant polysaccharides, are also shown to trigger biofilm formation on the root surface, and surfactin is not required for this colonization (16). However, experimental evidence showing root-specific regional preference by PGPR, including *Bacillus* spp., has not yet been elucidated.

Amino acid metabolism is closely linked to plant–microbe interactions, providing signaling molecules, nutrients, and defense compounds (18, 19). Among amino acids, L-Glutamate (Glu) administration has been known to modulate plant–microbe interactions; for example, Glu administration in rice plants triggers resistance against *Magnaporthe oryzae* and *Alternaria alternata* (20, 21). A recent study showed that Glu also works as a wounding signal that triggers long-distance defense signaling in a manner dependent on Glutamate-like receptor (GLR)3.3 and GLR3.6 (22). While the role of Glu in plant–benign microbe interactions has not been established, Glu supplementation in the growth media has been shown to induce surfactin production in *Bacillus mojavensis* A21 and *B. subtilis* 168 (23, 24). However, how supplementation of Glu may interplay with microbially derived metabolites, such as surfactin, is not established.

A root map was generated in tomato plants subjected to different surfactin and exogenous Glu treatments to further investigate the mechanisms underlying the Glu–surfactin interplay in root colonization by the surfactin-null *B. subtilis* strain (sfp^−^). To determine whether Glu and/or surfactin change root colonization and promote specific colonization patterns along the root surface, we manipulated surfactin biosynthesis in *B. subtilis* strains and examined the effects of exogenous Glu on both sfp-null and sfp-overproducing strains. In both experiments involving either manipulated surfactin biosynthesis or exogenous Glu, different patterns of root colonization were identified, which suggests that Glu is an effective root-colonizer inducer in tomato under hydroponic conditions.

## MATERIALS AND METHODS

### Seedling preparation

Tomato seeds (*Lycopersicon esculentum* cv. Amish Paste) were purchased from Johnny’s Select Seeds. Seeds were soaked in water for at least 20 min and then suspended and shaken in a 2:1 solution of commercial bleach to sterile water with a drop of TWEEN-20 for 20 min. Seeds were then shaken in 70% ethanol (EtOH) for 30 s and rinsed three times with sterile water. Seeds were placed on ½-strength Murashige and Skoog (MS) agar medium (25) and incubated under a photoperiod of 12-h day 12-h night cycle for 7 days at a photosynthetically active radiation (PAR) of ~100 µmol m^−2^ s^−1^.

### Culturing bacteria

*B. subtilis* strains used in this study included PY79 (a surfactin null producer lab strain, hereafter PY79 sfp^−^), PY79::mNeonGreen (fluorescent reporter strain), PY79 lacA::Pveg-sfp::cat (surfactin producer strain, hereafter PY79 sfp^+^), and UD1022 (a well-characterized PGPR strain used as control). Strains PY79 and UD1022 were cultured on LB media and incubated at 37°C. PY79::mNeonGreen and PY79 lacA::Pveg-sfp::cat were cultured on LB agar supplemented with kanamycin (5 µg mL^−1^) and chloramphenicol (5 µg mL^−1^), respectively. For plant incubation experiments, overnight cultures grown in liquid LB with appropriate antibiotics were used (see Table 1).

**TABLE 1:** List of bacterial strains used in the study

Bacterial strain	Antibiotic selection	Reference
*Bacillus subtilis* PY79 (sfp^−^)	No selection	Gift from Devon Stork, Ethan Garner, and George Church
*Bacillus subtilis* PY79::mNeongreen	Kanamycin 5 µg mL^−1^	This study
*Bacillus subtilis* PY79 lacA::Pveg-sfp::cat (sfp^+^)	Chloramphenicol 5 µg mL^−1^	This study
*Bacillus subtilis* UD1022	No selection	(26, 27)

### Surfactin analysis and creation of surfactin overproducing strain

#### Surfactin quantification

Surfactin quantification was done via high-performance liquid chromatography with an Agilent 1100 Infinity model with a Zorbax Eclipse Plus-C18 column with a guard column installed. To isolate surfactin peaks, an isocratic method of acetonitrile with 1% trifluoroacetic acid was used. A volume of 50 μL of sample was injected and run for 10 min, and absorbance was analyzed at 205 nm. Quantification was accomplished by comparing the peak areas of three major peaks in the commercial standard. Surfactin was purchased from Smolecule (San Antonio, TX, USA). Stocks were made by dissolving surfactin in deionized water and diluting as needed.

### Strain generation

Polymerase chain reaction (PCR) based DNA amplification was performed with KOD Xtreme Hot Start Polymerase. PCR primers were designed to have 20 bp of overlap for ligation via Gibson isothermal assembly. The assembled construct was PCR amplified before purification and then used for transformation. The gene encoding Sfp was purchased from the Bacillus Genus Stock Center (BGSCID: ECE221).

Transformation was done by inoculating 1 mL of modified competence (MC) media from plates. At 4 h, the DNA was added to 200 µL of cells and grown at 37°C for 2 more hours before plating on selective media. MC media was made at a 10× stock. The 10× stock has final concentrations of 1 M potassium phosphate, pH 7, 30 mM sodium citrate, 20% (wt/wt) glucose, 220 mg/mL ferric ammonium citrate, 1% casein hydrolysate, and 2% potassium glutamate. Aliquots were stored at −20°C. MC media was made for the transformation and supplemented with 3 mM MgSO_4_. Colonies were verified to have the DNA by colony PCR, where cells were suspended in 50 μL TE buffer (10 mM Tris-HCl, 1 mM EDTA, pH 8.0) +10 µm glass beads, then vortexed for 10 min, boiled for 30 min, vortexed for 10 min. One microliter was used as a PCR template. PCR primers were purchased from Integrated DNA Technologies (Coralville, IA, USA). KOD Xtreme Hot Start and trifluoroacetic acid were purchased from Millipore Sigma (Burlington, MA, USA). Acetonitrile was purchased from RICCA (Arlington, TX, USA).

### Inoculation for root maps

Seven-day-old tomato plants were taken from ½ strength MS plates and suspended in 1.9 mL of liquid media in each well of 12-well plates. *B. subtilis* strains grown in liquid with appropriate antibiotic selection were pelleted and resuspended in ½ MS liquid media. Plants grown in liquid ½ MS with 15 g L^−1^ sucrose were treated with 100 µL of the 10⁷ cells mL⁻¹ (OD_600_ = 0.20) suspension, delivering approximately 10⁶ cells per well in 12-well plates. Inoculated plants were incubated at 25°C and PAR of ~100 µmol m^−2^ s^−1^ with a 12:12 h photoperiod.

We also evaluated the effect of surfactin (Srf) and a known plant resistance inducer (PRI) L-Glutamate (Glu) priming on modulating root colonization. For this, 7-day-old seedlings were transferred into ½ MS liquid medium supplemented with either Srf (25 µg mL^−1^) or Glu (5 mM). After 48 h of priming, seedlings were inoculated with *B. subtilis* strains as described above. Surfactin was then either left in the media (unwashed) or plants were moved to new, fresh media not containing surfactin (washed) at the time of inoculation. Root colonization was evaluated at 72 h post-inoculation through microscopy and colony-forming unit (CFU) quantification.

### Staining

Plant roots post 72 h of incubation with the non-reporter strains (PY79 sfp^+^ and UD1022) were stained with calcofluor white (dilution 1:1,000) for 15 min and then with SYTO13 (dilution 1:1,000) for 30 s before being mounted for bioimaging. Calcofluor white was used to stain plant cell walls. SYTO13 channels were overlaid on brightfield channels to make non-reporter strains (PY79 sfp^+^ and UD1022) visually comparable to those of reporter strain PY79::mNeonGreen.

### Colony-forming unit quantification

Root samples were excised 72 h post-bacterial inoculation, and fresh weights were recorded. Non-inoculated plants served as untreated controls. Root samples were rinsed with sterile DI water and transferred to 2 mL tubes containing 1 mL sterile DI water. Root samples were then homogenized using sterile pestles and vortexed briefly. The homogenates were serially diluted 1:10 to 10^−4^ in sterile DI water. From each dilution, 100 µL was spread in triplicate on LB agar with or without antibiotics. Plates were incubated at 37°C for 16 h, and CFUs were counted and expressed as CFU g⁻¹ fresh root weight.

### Plant phenotyping

Tomato (cv. Amish Paste) plants were germinated and maintained in ½ MS medium. Seven-day-old seedlings (post-germination) were inoculated with either PY79 sfp^−^ or PY79 sfp^+^ strain at one of three inoculum densities (OD₆₀₀ = 0.0001, 0.001, or 0.01, corresponding to ~10⁴, 10⁵, and 10⁶ cells mL⁻¹, respectively). Inoculations (5 µL) were applied by pipette at a fixed distance from the root zone on ½-strength MS agar plates, with or without 5 mM L-glutamate. Non-inoculated plants served as controls, and plates were incubated at 25°C under a 12 h light/12 h dark photoperiod. Root phenotypes such as length of primary roots (PR), length of lateral roots (LLR), and number of lateral roots (NLR) were quantified after 7 days of incubation.

### Microscopy

Roots were imaged from the first lateral root down to the root tip using the Stellaris 8 tauSTED/FLIM Confocal Microscope. The LasX program was used to input scale bars and prepare images to be made into figures.

### Root measurements

Root sections were measured using ImageJ software and lif files of merged regions.

### Pellicle assay

For the pellicle assays, the overnight grown and plated bacterial cells were selected from a plate and placed in 50 mL Falcon tubes containing 10 mL of LB broth and appropriate antibiotics as listed in Table 1. Liquid cultures were incubated in a shaking incubator maintained at 37°C and set at 220 rpm for 24 h. Cultures were removed from the shaker and diluted and adjusted to an optical density of 0.2 at 600 nm (OD_600_) using LB medium, and 10 μL (for a targeted concentration of ~10^6^ cells/mL) were inoculated into 1 mL of MSgg (monosodium glutamate–glycerol) medium, the pellet culture assays were set in 24-well plates and were incubated at 25°C and 30°C without shaking. Different conditions, including exogenous supplementation of surfactin (25 µg mL^−1^) and glutamate (5 mM), were added to the MSgg medium. Pellicles were harvested and photographed at 72 h post-inoculation.

### Replication, data analysis, and statistics

All the experiments involving root colonization, root mapping, CFUs enumeration, and surfactin analysis were replicated twice with three biological replicates. For root mapping, three biological replicates were analyzed for microscopy. Micrographs were analyzed post-imaging for colonization patterns. Of the three images analyzed and then repeated, representative images were selected. For pellicle experiments, three biological replicates were used in separate wells and replicated twice. Representative images were selected, taking all biological and technical replicates into consideration. Primary root length, number, and length of lateral roots were measured using ImageJ. A one-way ANOVA was performed to determine the effects of differing bacterial concentrations, as well as a two-way ANOVA to observe the effect of two bacteria (one concentration), in the absence or presence of glutamate. A Tukey multiple comparison was calculated from the means using GraphPad Prism, at a significance level of *P* < 0.05.

## RESULTS

### Root colonization of a lab strain of *B. subtilis* (sfp^−^)

**Fig 1:**
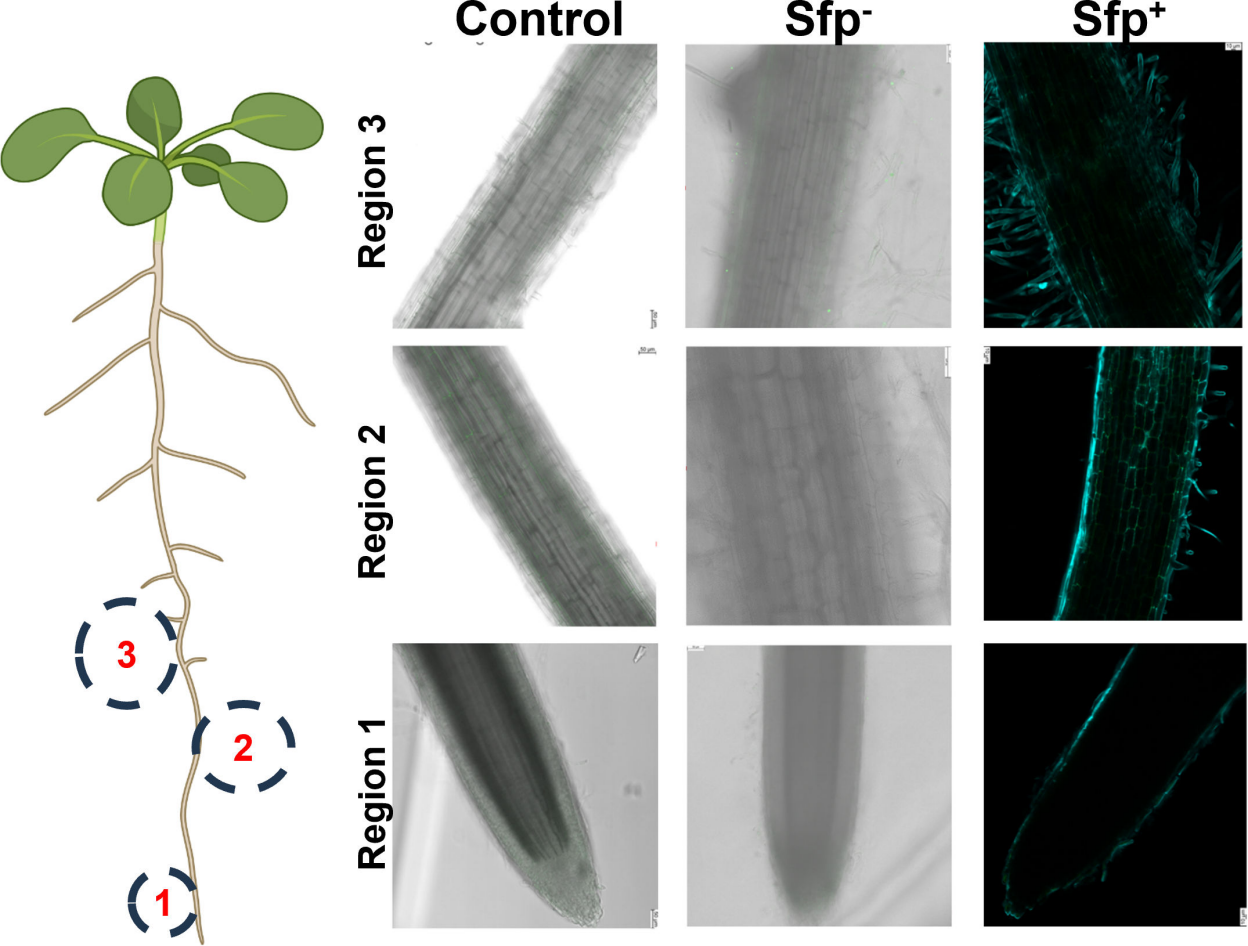
Root map of young tomato seedlings showing regions of root colonization by *B. subtilis* PY79::mNeonGreen sfp^−^ and PY79 sfp^+^ strains. Plant roots were imaged for the entire root map, but colonization was specifically monitored in the three root regions. Region 1: root tip; Region 2: elongation zone; Region 3: mature region with lateral root primordia. Surfactin null strain (sfp^−^) and surfactin overproducing strain (sfp^+^) were inoculated at 10^7^ cells mL^−1^. Sfp^+^ strain inoculated roots were stained with calcofluor white (cyan; root cell wall) and SYTO13 (green; bacteria). Plants were imaged 72 h post-inoculation with sfp^−^ and sfp^+^ strains. Untreated roots were used as a control. Scale bar = 5 mm.

To evaluate the specific root colonization patterns by different *B. subtilis* strains, a root map in young tomato seedlings was created (Fig. S1). The root maps were created to monitor preferred colonization in three different root regions, root tip (Region-1), elongation zone (Region-2), and maturation and lateral root region (Region-3) (Fig. S1). Tomato seedlings (7-day-old) grown *in vitro* were inoculated with various bacteria (sfp^−^ and sfp^+^), and different regions of roots were imaged finely for root-specific colonization. The entire roots were imaged starting from the root tip to the first lateral position (Regions 1–3) post-treatment (Fig. S1 and S2). Tomato roots inoculated with the surfactin-null strain PY79 sfp^−^ showed no colonization in any root region 72 h post-inoculation and appeared like the untreated control plant, which had not been treated with any benign strains of bacteria (Fig. 1).

### A surfactin overproducing strain is not a successful colonizer

Having shown that a surfactin null strain (sfp^−^) showed no to minimal colonization on younger tomato roots under a hydroponic system, an experiment was designed to manipulate surfactin biosynthesis in sfp^−^ strain background. To this end, a surfactin-overproducing strain (sfp^+^) was used to inoculate roots, and colonization data were imaged post 72 h of inoculation. We engineered a surfactin-producing PY79 (sfp^+^) by genome integration of the *sfp* gene under constitutive control by the P_veg_ promoter into the *lacA* locus. This PY79 sfp^+^ strain relies on the native *srf* operon and is under native regulation for the assembly of surfactin. Tomato plants supplemented with sfp^+^ strain didn’t show any colonization post 72 h of treatment (Fig. 1). A root map of the UD1022 was also created to compare how this undomesticated strain colonized the roots compared to the domesticated strains. UD1022 colonized both the mature region (Region-3) and, in some cases, the elongation zone (Region-2) as well (Fig. S3). In addition, UD1022 showed specific colonization in both mature and elongation regions compared to lab and surfactin-engineered strains (sfp^−^ and sfp^+^).

### Exogenously supplemented surfactin and its role in root colonization

**Fig 2:**
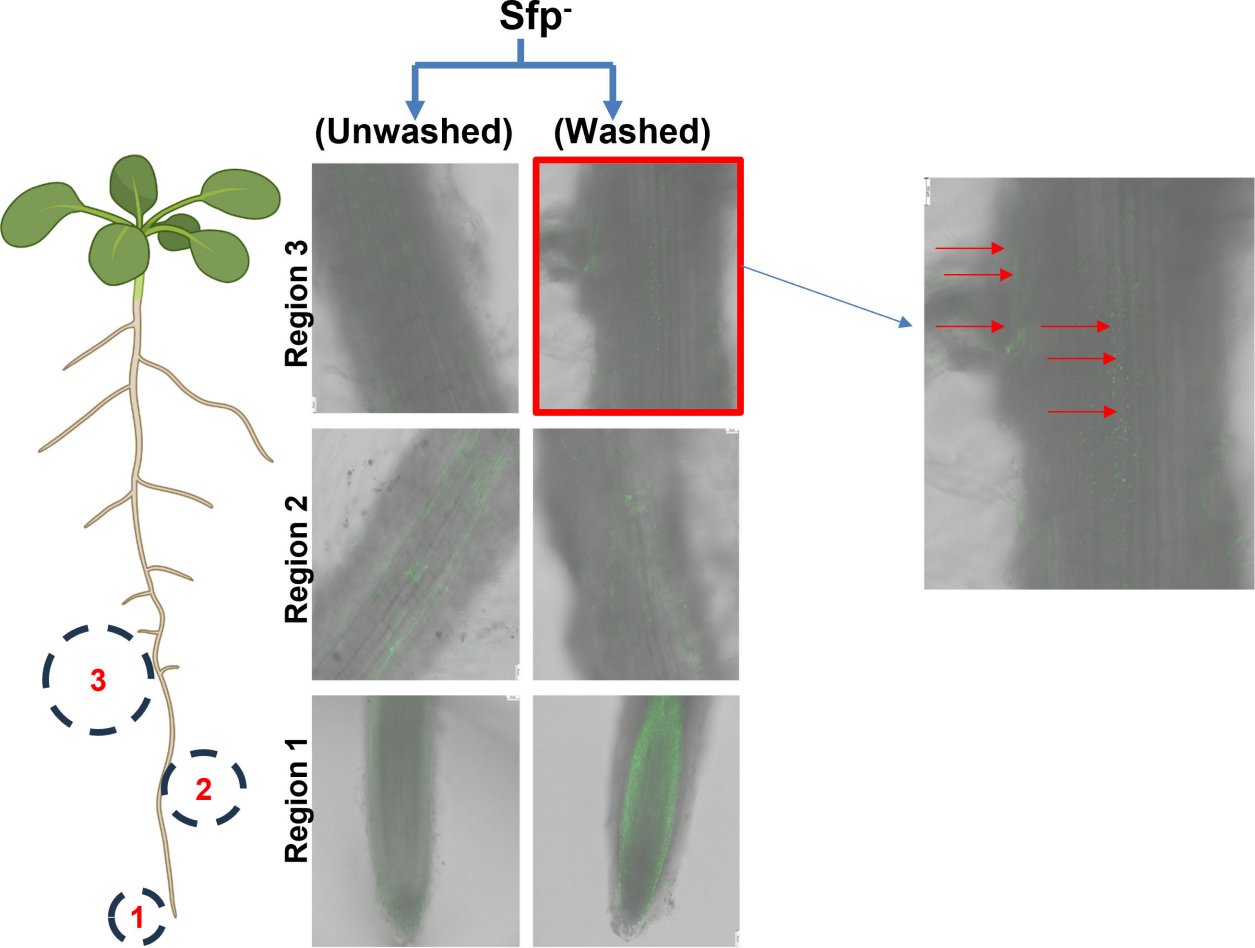
Exogenously supplemented surfactin and its role in root colonization of *B. subtilis* PY79::mNeonGreen (sfp^−^) on tomato roots. Plant roots were imaged for the entire root map, but colonization was specifically monitored in the three root regions. Region 1: root tip; Region 2: elongation zone; Region 3: mature region with lateral root primordia. Plants were primed with exogenous surfactin, inoculated with sfp^−^, and were imaged 72 h post-inoculation. Green fluorescence indicates PY79::mNeonGreen (sfp^−^) bacterial cells. Roots were not stained with calcofluor white. The bright green signal at the root tip region represents autofluorescence. The inset image shows bacterial colonization (arrows) in the mature region. Prior to imaging, roots were either washed or left unwashed of exogenous surfactin traces. Scale bar = 5 mm.

The inability of the surfactin-engineered strain (sfp^+^) to effectively colonize tomato roots made us postulate that an adequate concentration of surfactin may be required for the root colonization by *B. subtilis*. To observe its potential role in root colonization, surfactin was exogenously added to 1/2 MS medium to prime plants for 48 h prior to the addition of surfactin-null strain PY79 (sfp^−^). Reduced colonization was observed 72 h post-inoculation on both washed and unwashed plants by the sfp^−^ strain in the mature region (Region-3), with unwashed roots showing more colonization than washed plants. Colonization was observed on the mature regions of the root and only in these regions. Both the unwashed (Fig. 2) and washed (Fig. 2) conditions showed colonization in the mature regions (Region-3) of the root. The data suggest that the exogenously surfactin-supplemented roots facilitate root colonization by a surfactin-null strain PY79. In addition, the preferential colonization by the surfactin-null strain PY79 in the mature region (Region-3) of the root suggests an unknown conducive signaling favoring mature region colonization by the bacteria.

### Surfactin analysis in the overproducing strain

**Fig 3:**
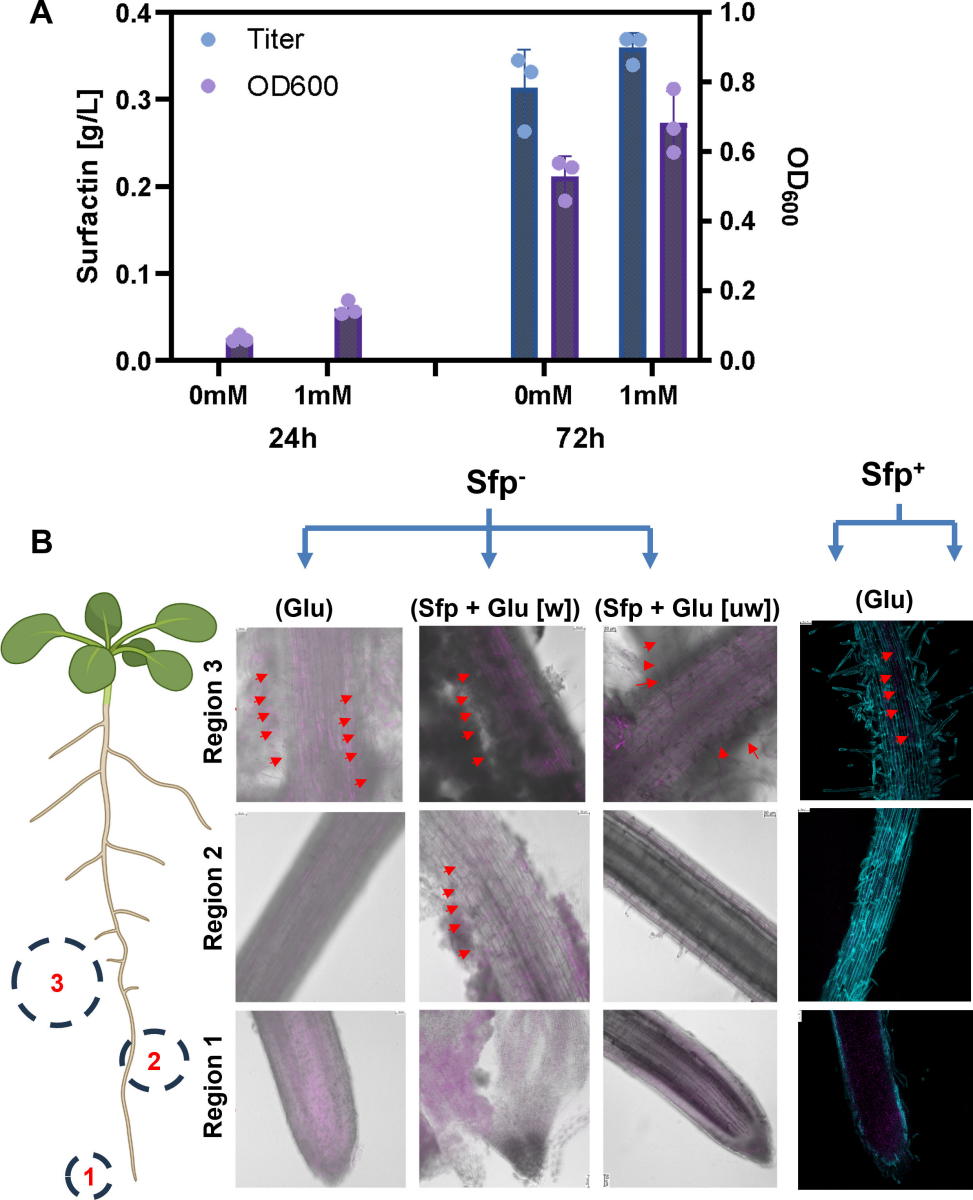
(**A**) Surfactin production and cell growth of *B. subtilis* PY79 sfp^+^ under static conditions, incubated at 25°C. Surfactin titers and cell growth of sfp^+^ strain were monitored post 1 mM glutamate supplementation under static conditions. Both surfactin titer and growth (OD_600_) were measured 72 h post-glutamate treatment. (**B**) Role of glutamate on root colonization in sfp^−^ and sfp^+^ strains. Plant roots were imaged for the entire root map, but colonization was specifically monitored in the three root regions. Region 1: root tip; Region 2: elongation zone; Region 3: mature region with lateral root primordia. Glutamate was added to the media at a concentration of 5 mM. The reporter strain PY79::mNeonGreen (sfp^−^) was imaged and pseudo-colored purple. The roots and non-reporter strains were fixed and stained with calcofluor white (cyan; root cell wall) and SYTO13 (pseudo-colored purple; bacteria). Glutamate-imbibed roots were primed with exogenous surfactin and inoculated with sfp^−^ and sfp^+^ strains. The dark color in the images with Glu represents bacterial colonization. The sfp^+^ strain-treated roots show colonization (arrows) in the mature region. Purple color (arrows) shows root colonization. Prior to imaging, roots were either washed or unwashed of exogenous glutamate/surfactin traces. Scale bar = 5 mm.

We showed that the sfp^+^ strain failed to colonize the tomato roots, suggesting the conditional specific response of surfactin in root colonization. To evaluate if the sfp^+^ strains were able to biosynthesize surfactin, we tested the growth and production of the sfp^+^ strain under static incubation at 25°C (Fig. 3A). Engineered strains were also able to grow similarly in both rich and minimal media (Fig. S4). In addition, the strain morphology of the engineered strain was similar to the parental strains (Fig. S4). The data showed that sfp^+^ strain was able to biosynthesize surfactin at 72 h of incubation (Fig. 3A).

### Glutamate assists with the root colonization in PY79 species

To analyze the role of L-Glutamate in surfactin interplay for root colonization phenotype, tomato roots were treated with glutamate (5 mM). Exogenous treatment of Glu to tomato roots with surfactin-null strain (sfp^−^) showed enhanced root colonization in the mature region of the roots (Fig. 3B; Fig. S2). Colonization was primarily observed within the mature region of the root in both sfp^−^ and sfp^+^ and does not extend to either the zone of elongation or the root tip (Fig. 3B). A similar trend also appeared to occur with sfp^+^, wherein surfactin overproducing line showed root colonization in the mature region with exogenous treatment of Glu (5 mM) (Fig. 3B). This data suggests that the addition of Glu may trigger root-specific colonization in the mature region (Region-3) independent of surfactin.

### Glutamate supplementation is not needed for surfactin biosynthesis in static bacterial cultures

Glu supplementation triggered root colonization in sfp^+^ strains. Surfactin biosynthesis was favored under static culture conditions with or without supplementation of 1 mM L-Glu (Fig. 3A). Under these conditions, growth and surfactin production were comparable over the 72-h period in both conditions (Fig. 3A). These data indicate that Glu supplementation may not directly interface with surfactin biosynthesis, at least under static culture conditions.

### Priming roots with Glu and surfactin triggers root colonization by PY79

Having shown that plants treated with exogenous Glu and inoculated with surfactin null and overproducing strains showed similar root colonization phenotypes, we evaluated the role of exogenous surfactin in the Glu-surfactin interplay for the root colonization phenotype. When plants were primed with both Glu and surfactin prior to inoculating roots with the surfactin null strain sfp^−^, this resulted in colonization in multiple regions of the roots. This colonization occurred in both washed and unwashed conditions at a concentration of 5 mM of Glu. Addition of Glu (5 mM) did not alter the plant or microbial growth (data not shown). Interestingly, plant root cultures that were subjected to wash treatment showed less colonization than that of the unwashed Glu treatments (Fig. 3B). Contrary to the mature region-specific colonization of surfactin-treated roots, roots treated with Glu and surfactin together colonized the entire roots and did not show specificity to any region in particular (Fig. 3B). The phase image of the entire root map also showed the entire root colonization by surfactin-null strain (sfp^−^) treated with exogenous surfactin and Glu (Fig. S4).

### Glutamate supplementation induces surfactin biosynthesis in plant-*B. subtilis* cultures

We tested the surfactin biosynthesis in tomato plants inoculated with surfactin overproducing strain (sfp^+^) supplemented with L-Glu (5 mM). Surfactin overproducing strain (sfp^+^), when cultured with the tomato plant and 5 mM glutamate and extracted for surfactin, showed the presence of surfactin as the standard surfactin HPLC traces (Fig. S5). As expected, neither these plants nor plants inoculated with PY79 (sfp−) showed the presence of surfactin peaks (Fig. S5). Contrary to the bacterial static culture surfactin analysis with L-Glu, the plant colonized sfp^+^ strain with exogenous surfactin showed traces of surfactin, suggesting that L-Glu may elicit surfactin biosynthesis in the sfp^+^ strain during root colonization.

### Root colonization quantification via CFU enumeration

**Fig 4:**
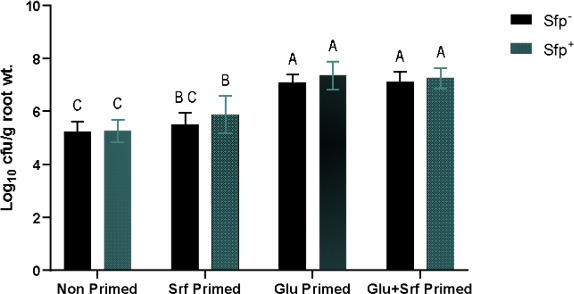
Log_10_ CFU per gram root fresh weight 72 h post-inoculation under different priming treatments. Priming treatments included non-primed, surfactin (Srf) primed, glutamate (Glu) primed, and surfactin + glutamate (Glu+Srf) primed media. Plants were inoculated with sfp^−^ and sfp^+^ (100 µL of 10^7^ CFU/mL). Each bar represents the mean (*n* = 18) with error bars showing standard deviation (SD). Means followed by different letters are significantly different (Tukey’s HSD; *P* < 0.05).

The effect of exogenous priming on root colonization potential of sfp null (sfp^−^) and sfp overproducing (sfp^+^) strains was evaluated 72 h post-inoculation. Each well was initially inoculated with 10⁶ cells, and CFU counts were expressed as log₁₀ CFU per gram root fresh weight after 72 h. CFU counts varied significantly across *Bacillus* strains (sfp⁻ and sfp^+^) and priming conditions (non-primed, surfactin primed, Glu primed, and Glu + surfactin primed). CFU enumeration was performed by plating 0.1 mL per dilution, with countable plates defined as those having 35–250 colonies. Non-inoculated control samples, plated at 10⁰ dilution, had CFU counts below the threshold for reliable quantification (<35 colonies). Significant differences were observed among the treatments (*P* < 0.05, Tukey’s HSD), indicating that both priming and bacterial strain influenced root colonization (Fig. 4). Both sfp⁻ and sfp^+^ strains showed the lowest colonization in non-primed plants with statistically similar CFU counts (~5.2 log₁₀ CFU/g) (Fig. 4). Whereas surfactin-primed roots showed a moderate increase in colonization for both sfp^+^/sfp^−^ strains. Glutamate priming resulted in significantly higher root colonization compared to both non-primed and surfactin-primed treatments, with no significant difference between the two strains. The wild *B. subtilis* (UD1022) isolate showed more colonization compared to both sfp^+^/sfp^−^ strains under unprimed conditions (Fig. S6). Dual priming with Glu and surfactin also led to similarly high colonization levels, with sfp⁻ and sfp^+^ strains performing comparably (Fig. 4; Fig. S6), suggesting that Glu priming may override surfactin-dependent *Bacillus* root colonization.

### Glutamate aids in the development of thicker pellicles

Having shown that Glu and surfactin exogenous application to tomato roots changed the root colonization pattern, we evaluated the role of Glu in *B. subtilis*’ ability to form pellicles on abiotic surfaces. In addition, pellicle experiments were conducted in parallel with the dual temperatures of 30°C and 25°C (room temperature) to observe if temperature had any impact on pellicle formation. Abiotic surfaces appear to display different results from the biotic surfaces seen in the root maps, specifically in the cases of PY79 and sfp^+^ strain. Both surfactin overproducing strain (sfp^+^) and biocontrol strain UD1022 formed continuous pellicles independent of Glu/surfactin or temperature conditions (Fig. S7). Interestingly, biocontrol strain UD1022 formed wrinkled pellicles under Glu treatments compared to the lab strains (Fig. S7). There was also a pigment change in both sfp^+^ and UD1022 at 30°C (Fig. S7). While this change only occurred in glutamate treatments for sfp^+^ strain, UD1022 appeared to turn a darker yellow at 30°C in all treatments. The data suggest pellicle formation in *B. subtilis* is independent of Glu and surfactin treatment compared to Glu-dependent biotic surface colonization.

### Role of surfactin and glutamate on root phenotype

To analyze the role of surfactin and glutamate supplementation with tomato plants, plants were treated with sfp^−^ or sfp^+^ and grown with different bacterial inoculum densities. Plants post-incubation were analyzed for any root phenotype in terms of length of PR, NLR, and LLR. Though the plants treated with higher inoculum of sfp^−^ showed reduced LRR, they were not significantly different from lower inoculum densities (Fig. S8A and B). The sfp^+^ strain showed an inverse LRR phenotype in the lower density treatment with reduced LRR, compared to the higher inoculum, but was not statistically different than the other treatments (Fig. S8A and B). The data showed that supplementation of glutamate to tomato plants altered the colonization patterns, which were surfactin independent. The root phenotyping data showed that there were no significant changes to the LLR and NLR phenotype post-glutamate supplementation. Surprisingly, plants exposed to glutamate showed a decrease in LLRs (Fig. S9). Plants treated with glutamate and srf ^+^ showed significantly increased LLR compared to the sfp^−^ strain (Fig. S9).

## DISCUSSION

To protect crops from plant pathogens to reduce disease incidences, we need to better understand the role of PGPR in the soil that interact with plants and plant–pathogenic microbe interactions. *Bacillus* spp. are commonly found in disease-suppressive soils and are often used in biocontrol methods to help prevent disease across various crops (1, 28). *B. subtilis* is often studied as it is known to not only prevent disease but promote plant growth as well (2). The interaction of PGPR, particularly *Bacillus* spp., with plants is mediated through root exudates (29). Though much attention has been focused on how root exudates influence the soil and rhizospheric microbiome, the impact of root region-specific exudations and their role in root colonization by PGPR is not known. It is shown in many plant species that the root secretions modify rhizosphere colonization by PGPR, but how different regions of roots modulate secretion profile while interacting with rhizobacteria may shed more light on preferential colonization pattern of PGPR.

Surfactin is known to regulate critical microbial functions, including antibiosis, against major bacterial species (30). The role of surfactin in modulating plant–microbe interactions has been questioned and debated. Some reports suggest that surfactin is an important metabolite that regulates root colonization and disease reduction against pathogens in plants (31). In contrast, Thérien et al. (32) reported that surfactin may not play a critical role in root colonization. The gaps in the literature also do not clarify the specific role of plants in mediating surfactin-dependent colonization. For example, at this juncture, we do not know which region of the root prefers PGPR colonization and if root-specific colonization of *Bacillus* spp. is surfactin dependent. In addition, the *in vivo* surfactin production by *B. subtilis* on the root surface during the process of colonization is also not known. Our data show that the mature region of the root is preferred by *B. subtilis* during hydroponic culturing at 25°C. Contrary to the literature, we showed that *B. subtilis,* irrespective of bacterially derived surfactin, prefers to colonize at the mature region of the roots. The mature region of the roots is an important rhizoplane frontier and initiates the formation of lateral primordia (33) and may secrete specific metabolites, which could trigger *Bacillus* colonization. Incidentally, a recent study demonstrated that *Pseudomonas protegens* CHA0 preferentially colonizes the mature region of plant roots (near lateral root primordia) due to glutamine secretion associated with vascular development (34). Both the recent work and our study suggest that colonization in the mature root region may have functional significance, potentially promoting the preferential root colonization of the beneficial microbes in plants. Interestingly, our data showed that microbially derived surfactin may not be sufficient for root–microbe interaction initiation and colonization, and an exogenous level of surfactin may be needed to trigger colonization. This was shown when an exogenous surfactin was added to the surfactin-null strain PY79 (sfp^−^), leading to root colonization in the mature region.

However, under abiotic conditions, we noted that sfp^+^ formed pellicles similar to those of the biocontrol strain UD1022, while PY79 was unable to form a complete and continuous pellicle. This aligns with the findings of Stoll et al. (1), wherein it was shown that the production of surfactin assists in the formation of biofilms and microcolony initiation. However, the production of surfactin or lack thereof was not the sole determinant of whether a microbe would be a successful colonizer or not (1). Other studies have also noted that even without surfactin, some *Bacillus* species are still able to form weak biofilms if they are able to produce NRP, such as an iturin or bacillomycin (32). One of the links to surfactin’s role in biofilm production is the linkage to the activation of the gene *spo0A,* which produces a response regulator protein (35). Even with a knockout of surfactin, many species were still able to produce a pellicle despite the changes in morphology and loss of the ability to float (35). A recent study showed that surfactin may be involved in triggering the rate of pellicle formation (36). The study shows that supplementation of surfactin may enhance the cell density in advancing pellicle timing (36). The study also indicates that surfactin biosynthesis may vary in different *B. subtilis* strains as the pellicle timing is a critical factor in the formation of biofilms on abiotic surfaces. Similarly, our data also showed that supplementation of surfactin did not alter pellicle formation in the surfactin-null strain PY79 (sfp^−^). The surfactin overproducing strain sfp^+^ showed a continuous pellicle formation compared to the surfactin null strain sfp^−^, suggesting that microbial surfactin production may help in pellicle formation on abiotic surfaces. The ability of a biocontrol strain UD1022 to form pellicle was like the sfp^+^ strain, suggesting that surfactin levels in both strains may be similar. Both sfp^+^ and biocontrol strains showed similar pellicle formation under all the treatments at two different incubation temperatures (25°C and 30°C). These data suggest that surfactin-mediated pellicle formation is independent of the incubation temperature conditions in our studies. Most reports showing pellicle formation in the literature use 30°C as the incubation temperature as a conducive condition for bacterial growth (37). The idea to test pellicle formation as a factor of surfactin production under a lower temperature was to mimic the root colonization studies with *B. subtilis* and plant hosts. The effect of surfactin and its role in both biotic and abiotic surface colonization in a gnotobiotic system, either for root association or for pellicle formation, are difficult to tease out the underlying mechanisms; thus, future experiments should include gene expression coupled with microscopy to assess the effect of specific root zones in triggering root colonization at a single-cell level.

The amino acid glutamate (Glu) is an important signaling molecule in plants, playing roles in many important functions, including wound response, pathogen resistance, and response/adaptation to abiotic stress (38). Glutamate is released from the maize roots under duress from iron (Fe) deficiencies in the soil (39). In addition, glutamate has been shown to trigger a Ca^2+^ transient-based systemic signaling response against herbivory (22). Glutamate also plays a role as a PRI-activating plant defense against pathogenic fungi (40). However, studies regarding the impact of glutamate in the rhizosphere to initiate root colonization or signaling with PGPR are lacking. The role of glutamate to either encourage or deter colonization of roots appears to be unknown. In our study, we observed an increase in the root colonization when plants and bacteria were exposed to glutamate exogenously and when plants were primed with glutamate. The data clearly showed that supplementation of glutamate overrides surfactin for root colonization even in a surfactin null strain. The data showing the role of exogenous Glu on surfactin titers in static bacterial culture/plant-associated sfp^+^ strain dictates that surfactin biosynthesis and Glu may be operating differently for bacterial colonization and are condition-specific responses. PGPR respond not only to biotic stressors like disease, but also to abiotic stressors as well (41). Since increased bacterial root colonization was observed when glutamate was added to the media, it could be possible that the microbes were responding to a perceived abiotic stressor occurring to the plant and following the signal to colonize to help mitigate said stress. In accordance, Kim et al. (42) also indicated that internal levels of glutamic acid within the plant are important in the cultivation of beneficial microbial communities. The role of glutamate in triggering pellicle formation on the abiotic surface in both sfp^−^ and sfp^+^ strains, as well as non-glutamate treatments. The data show that glutamate plays a critical role in triggering root colonization compared to its role in facilitating pellicle biofilm formation on an abiotic surface. The role of root surface chemistry and exudation from different root zones may be critical for bacterial colonization. It is known that benign microbes, including *B. subtilis,* induce innate root defense responses (26). Benign microbes, including *Bacillus* species, have evolved to suppress plant root defense response to initiate colonization and biofilm formation. The role of surfactin and/or glutamate in suppressing root defense is not established. Arguably, it will be interesting to conceptualize that different root regions, such as root tip, central elongation zone, or mature region, may have a different root surface/secretion chemistry, which may change the pattern of colonization by beneficial bacterial species (Fig. S10). Detailed studies are needed to decipher the role of glutamate (either plant-derived or soil-based) for colonization on both abiotic and biotic surfaces and for evaluating its role in modulating innate root defense response and secretion/surface chemistry.

### Conclusion

PGPR are vital for plant growth and health; however, many of the mechanisms of how and where they colonize the plant roots are largely unknown. To explore this largely unexplored area of science more thoroughly, we created detailed root maps of a young tomato plant. Through the creation of the root map, we were able to observe that most of the PGPR colonization appears to occur around the mature region of the root and not around the root tip or central elongation zone. This trend could be due to specific exudates released from the plant around this area or due to the larger surface area, as colonization was also observed around the root hairs. However, the presence or absence of microbially derived surfactin did not seem to modulate root colonization. The addition of glutamate to the plant growth environment challenged the pattern of colonization, especially when plants were primed with glutamate prior to the addition of PGPR. While microbes still colonized the mature region, in some cases, they also colonized the root tip and central elongation zone. The addition of surfactin to the media alone did not change these colonization patterns and only appeared to enhance colonization slightly. However, when glutamate was added to the media, colonization of the plant by the surfactin-null strain PY79 (sfp^−^) increased dramatically. Abiotic surfaces of the pellicle experiments yielded different results from the biotic surfaces of the roots. Both a biocontrol strain UD1022 and an sfp^+^ formed pellicles under various conditions. UD1022 even managed to form wrinkles and continuous biofilm at 22°C. This differed from what was observed in experiments involving root surfaces with the PY79 treatment. While PY79 could not colonize root surfaces on its own, once glutamate or surfactin (or both) was used as a treatment, colonization could be observed. Under abiotic conditions, PY79 was unsuccessful in forming continuous pellicles in the treatments. It is fair to conclude that pellicle formation or lack thereof may not be representative of the potential of a microbe to colonize biotic surfaces, such as a root. Overall, surfactin production by PY79 didn’t impact root colonization in tomatoes, and other microbially derived secondary metabolites should be investigated further. The role of surfactin to trigger root colonization may depend on the concentration of surfactin. In addition, the role of glutamate to trigger a surfactin-independent root colonization opens areas to verify and test other PRIs and amino acid derivatives to facilitate benign plant–microbe interactions. In addition, how glutamate modulates plant innate defense response and secretion chemistry to enhance root colonization needs to be studied at the mechanistic level.

## SUPPLEMENTAL MATERIAL

Supplemental figure legends spectrum.03181-25-s0001.docx Legends for Figure S1 to S10.

Supplemental figures spectrum.03181-25-s0002.pdf Figures S1 to S10.

ASM does not own the copyrights to Supplemental Material that may be linked to, or accessed through, an article. The authors have granted ASM a non-exclusive, world-wide license to publish the Supplemental Material files. Please contact the corresponding author directly for reuse.
