# Supplementary material for: Glutamate facilitates root colonization by plant growth-promoting rhizobacteria Bacillus subtilis in tomato seedlings
Source: Microbiol Spectr. 2026 Mar 13;14(4):e03181-25. doi: 10.1128/spectrum.03181-25 (PMC13055282; doi:10.1128/spectrum.03181-25)
Supplement: Supplemental figure legends — Legends for Figure S1 to S10. [file spectrum.03181-25-s0001.docx]

**Legends to the supplementary figure:**

**Supplementary Figure 1:** Schematic showing creation of a root map model for a young tomato seedling to evaluate root association pattern by *B. subtilis*. Plant roots were imaged post inoculation by PGPR (*B. subtilis* sfp strains, 100 µL of 10^7^ CFU/mL) for the entire root map, but colonization was specifically monitored in the three root regions. Region 1: Root tip; Region 2: Elongation zone; Region 3: Mature region with lateral root primordia.

**Supplementary Figure 2:** Root map showing the entire length of roots of a young tomato seedling. Plant roots were imaged post inoculation by PGPR (*B. subtilis* sfp strains, 100 µL of 10^7^ CFU/mL) for the entire root map, but colonization was specifically monitored in the three root regions. Region 1: Root tip; Region 2: Elongation zone; Region 3: Mature region with lateral root primordia (LRP). The micrograph shows root map of tomato seedings treated with sfp^-^ and sfp^-^ with exogenous glu and surfactin. The Regions I-III clearly shows significant colonization by sfp^-^ strain in Glu treated plants. Scale bar= 5mm. The bacteria treated roots were stained with calcofluor white. The dark circles (in red) in sfp-/Glu treatment shows bacterial colonization in Mature regions.

**Supplementary Figure 3:** Tomato roots were also inoculated with a biocontrol *B. subtilis* strain (UD1022, 100 µL of 10^7^ CFU/mL). Colonization was specifically monitored in the three root regions. Region 1: Root tip; Region 2: Elongation zone; Region 3: Mature region with lateral root primordia (LRP). The UD1022 roots were treated with SYTO13 (pseudo colored purple). The inset shows bacterial colonization (arrows) in the mature region of the root. Scale bar= 5mm.

**Supplementary Figure 4 A-H:** Growth of PY79, PY79 sfp^+^, and PY79::mNeonGreen in rich LB media (A) and ½ MS media supplemented with 1.5% sucrose and 10 mM glutamate (B). Colony morphology of PY79 sfp^-^ (C), PY79 sfp^+^ (D), and PY79::mNeongreen (E) on LB plates and PY79 sfp- (F), PY79 sfp+ (G), and PY79::mNeongreen (H) on ½ MS agar plates supplemented with 1.5% sucrose and 10 mM glutamate.

**Supplementary Figure 5:** HPLC traces of surfactin analysis in sfp^-^ and sfp^+^ strains treated with/without glutamate in a plant mesocosm suspension culture.

**Supplementary Figure 6:** Log₁₀ colony forming units (CFU) per gram root fresh weight 72 hours post-inoculation under different priming treatments. Priming treatments included non-primed, surfactin (Srf) primed, glutamate (Glu) primed, and surfactin + glutamate (Glu+Srf) primed media. Plants were inoculated with sfp^-^, sfp^+^, and UD1022 (100 µL of 10^7^ CFU/mL). Each bar represents the mean (n = 18) with error bars showing standard deviation (SD). Means followed by different letters are significantly different (Tukey’s HSD; p < 0.05).

**Supplementary Figure 7:** Effect of temperature, glutamate, and surfactin on pellicle formation in lab and a biocontrol *B. subtilis* strains. A) Pellicle formation of surfactin null strain (PY79) (sfp^-^), surfactin overproducer (sfp^+^) and a wild *B. subtilis* strain (UD1022) grown at 25^o^C. B) Pellicle formation of surfactin null strain (PY79) (sfp^-^), surfactin overproducer (sfp^+^) and a wild *B. subtilis* strain (UD1022) grown at 30^o^C. Different conditions including exogenous supplementation of surfactin (25 µg ml ^-1^), and glutamate (5 mM) were added to the media. Images of the pellicle were photographed on 72-hrs post inoculation.

**Supplementary Figure 8A-B:** The panel shows tomato root phenotypes with sfp^-^ and sfp^+^ strains. Different bacterial densities (5 µL of ~10⁶ to 10⁴ CFU/mL)) were applied to tomato plants (A), and plants post-treatment were recorded for root traits, such as length of primary roots (PR), length of lateral roots (LLR) and number of lateral roots (NLR) were quantified post 7 days of incubation (B). Letters indicate statistical similarity between the bacterial concentrations using one-way ANOVA and Tukey multiple comparisons (p-value < 0.05).

**Supplementary Figure 9A-D:** The panel shows tomato root phenotype with sfp^-^ and sfp^+^ treatment with/without glutamate (5mM) (A). Tomato plants were inoculated with sfp^-^ and sfp^+^ strains (~5 µL of 10⁶ CFU/mL). Plants post-treatment was recorded for root traits, such as length of primary roots (PR) (B), length of lateral roots (LLR) (C) and number of lateral roots (NLR) (D), which were quantified after 7 days of incubation. Two-way ANOVA was performed and Tukey multiple comparisons to determine statistical significance (p-value < 0.05). * = 0.0303, *** = 0.0008, **** = < 0.0001.

**Supplementary 10:** Schematic shows the root surface chemistry defining association of benign microbes on plant roots. The root zones such as root tip (Region-1) and central elongation zone (CEZ) (Region 2) are speculated to be preferred zones of an association by benign microbes compared to the mature region (MR) (Region 3) of the roots. The exudate chemistry is speculated to be different in zone *a-c* from root tip (c) to CEZ (b) to the mature region (a) of the root. It is conceptualized that these three regions (a-c) may have a different root surface and secretion chemistry, which may change the pattern of colonization by the beneficial bacterial species. In addition, surfactin production by *B. subtilis* species may be differential based on its association with the roots. Glutamate is shown to override innate defense response of plant roots for colonization phenotype which is independent of surfactin production.
