# Supplementary material for: Glutamate facilitates root colonization by plant growth-promoting rhizobacteria Bacillus subtilis in tomato seedlings
Source: Microbiol Spectr. 2026 Mar 13;14(4):e03181-25. doi: 10.1128/spectrum.03181-25 (PMC13055282; doi:10.1128/spectrum.03181-25)

SOM Figure 1

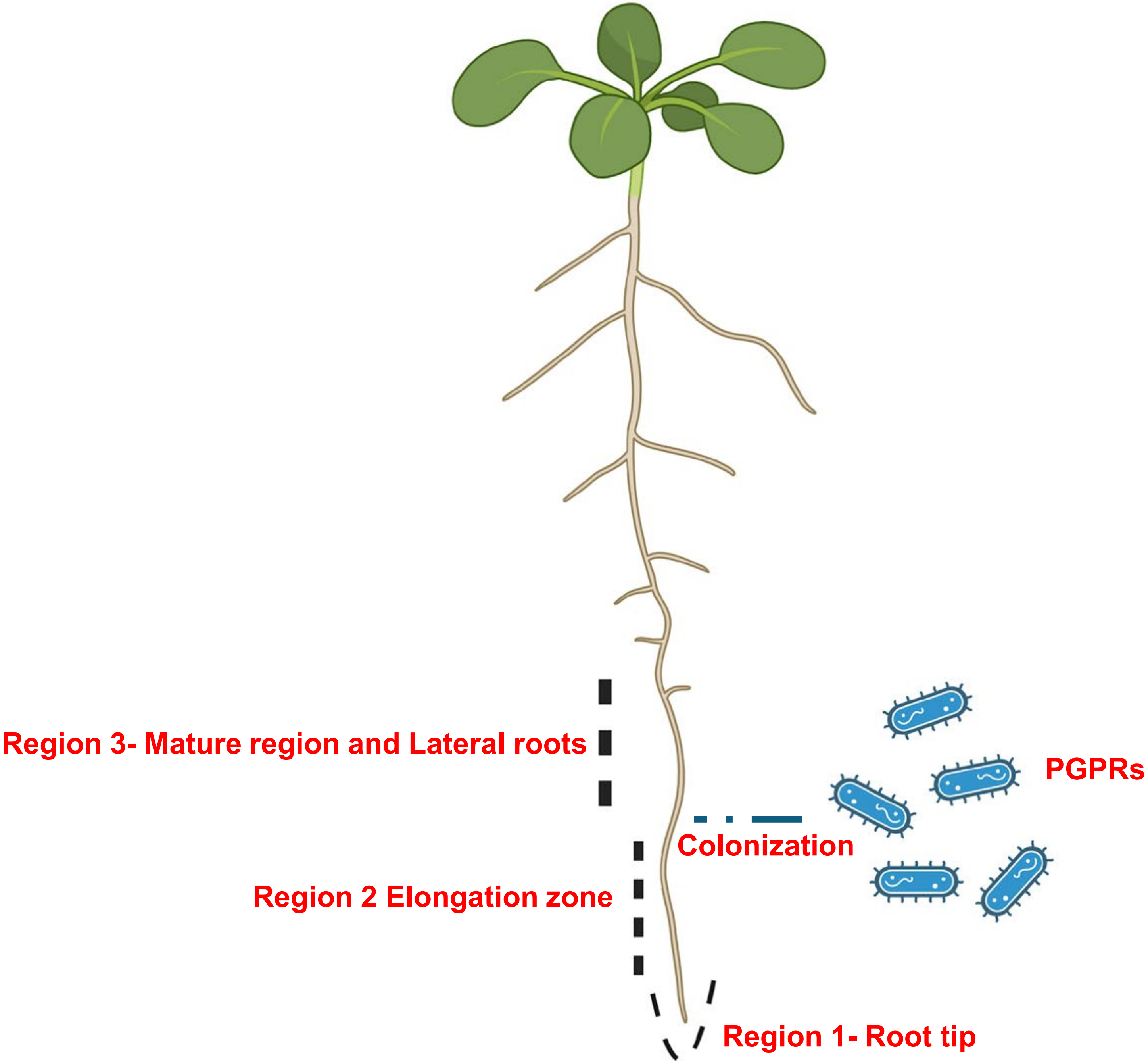
Region 3- Mature region and Lateral roots
Colonization
PGPRs
Region 2 Elongation zone
Region 1- Root tip

SOM Figure 2

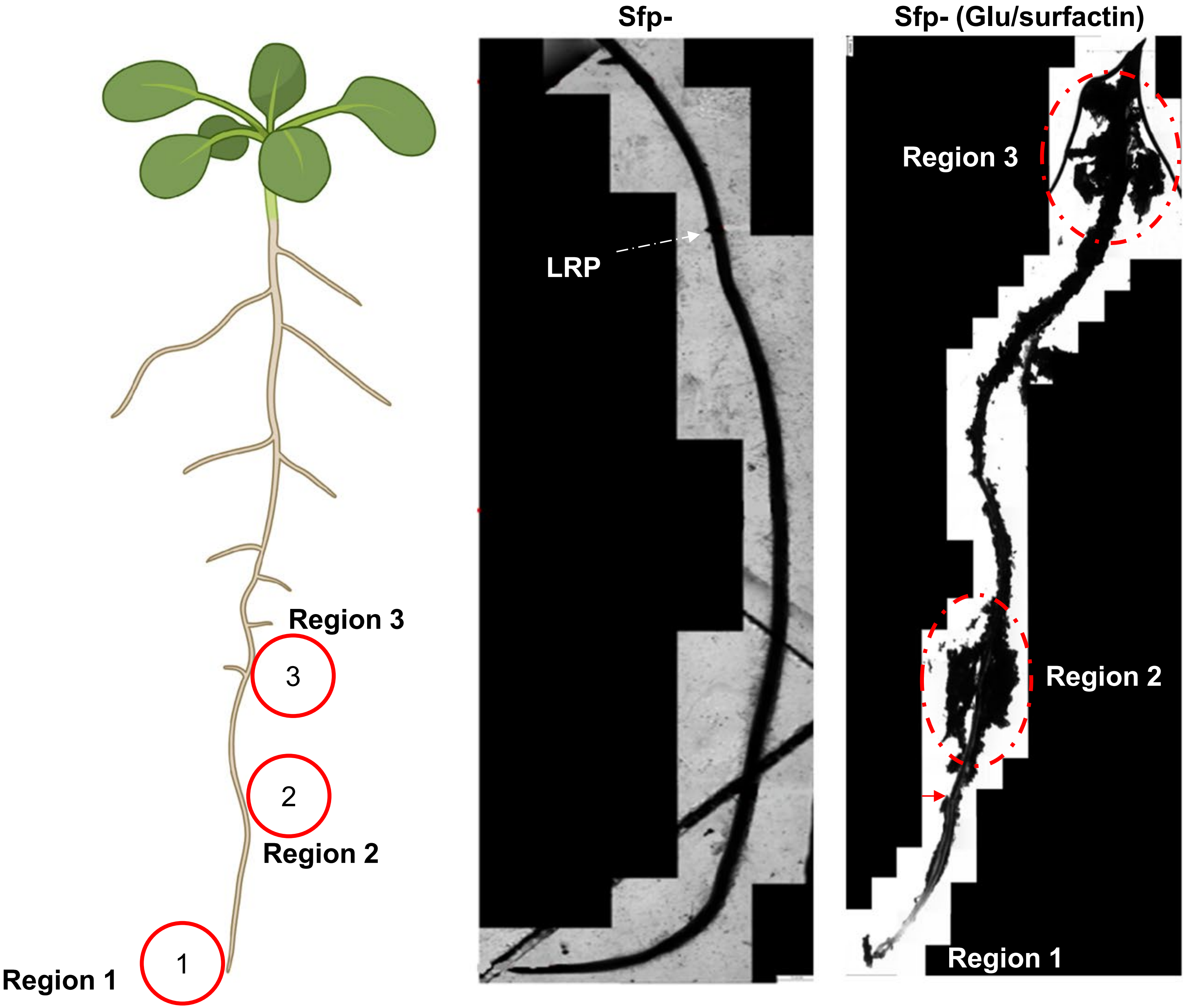
Region 3
3
2
Region 2
1
Region 1
Sfp-
LRP
Sfp- (Glu/surfactin)
Region 3
Region 2
Region 1

SOM Figure 3

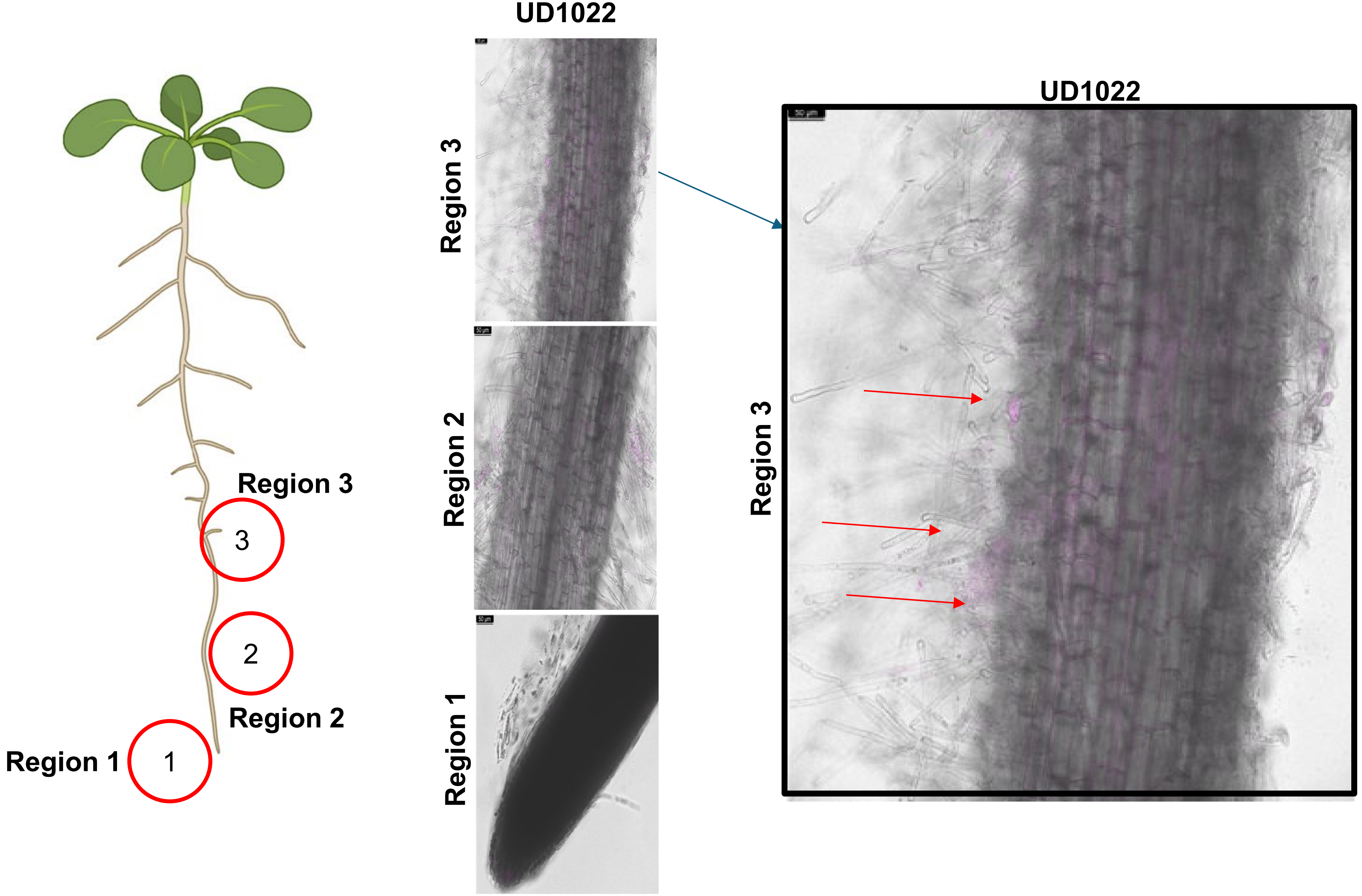
Region 3
Region 2
Region 1
3
2
1
UD1022
Region 3
Region 2
Region 1
UD1022
Region 3

SOM Figure 4

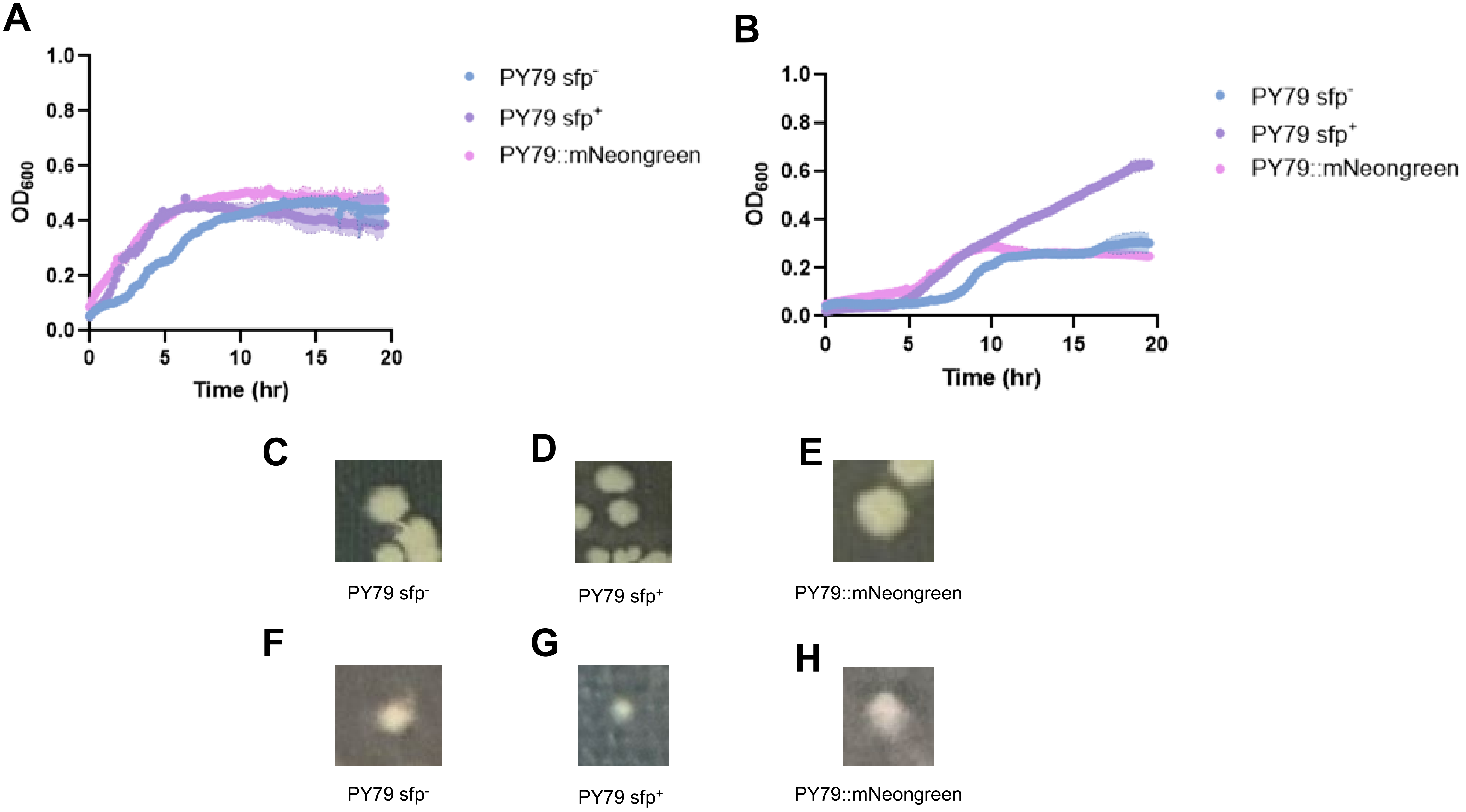
A
1.0
0.8
0.6
0.4
0.2
0.0
OD600
0
5
10
15
20
Time (hr)
PY79 sfp-
PY79 sfp+
PY79::mNeongreen
B
1.0
0.8
0.6
0.4
0.2
0.0
OD600
0
5
10
15
20
Time (hr)
PY79 sfp-
PY79 sfp+
PY79::mNeongreen
C
PY79 sfp-
D
PY79 sfp+
E
PY79::mNeongreen
F
PY79 sfp-
G
PY79 sfp+
H
PY79::mNeongreen

SOM Figure 5

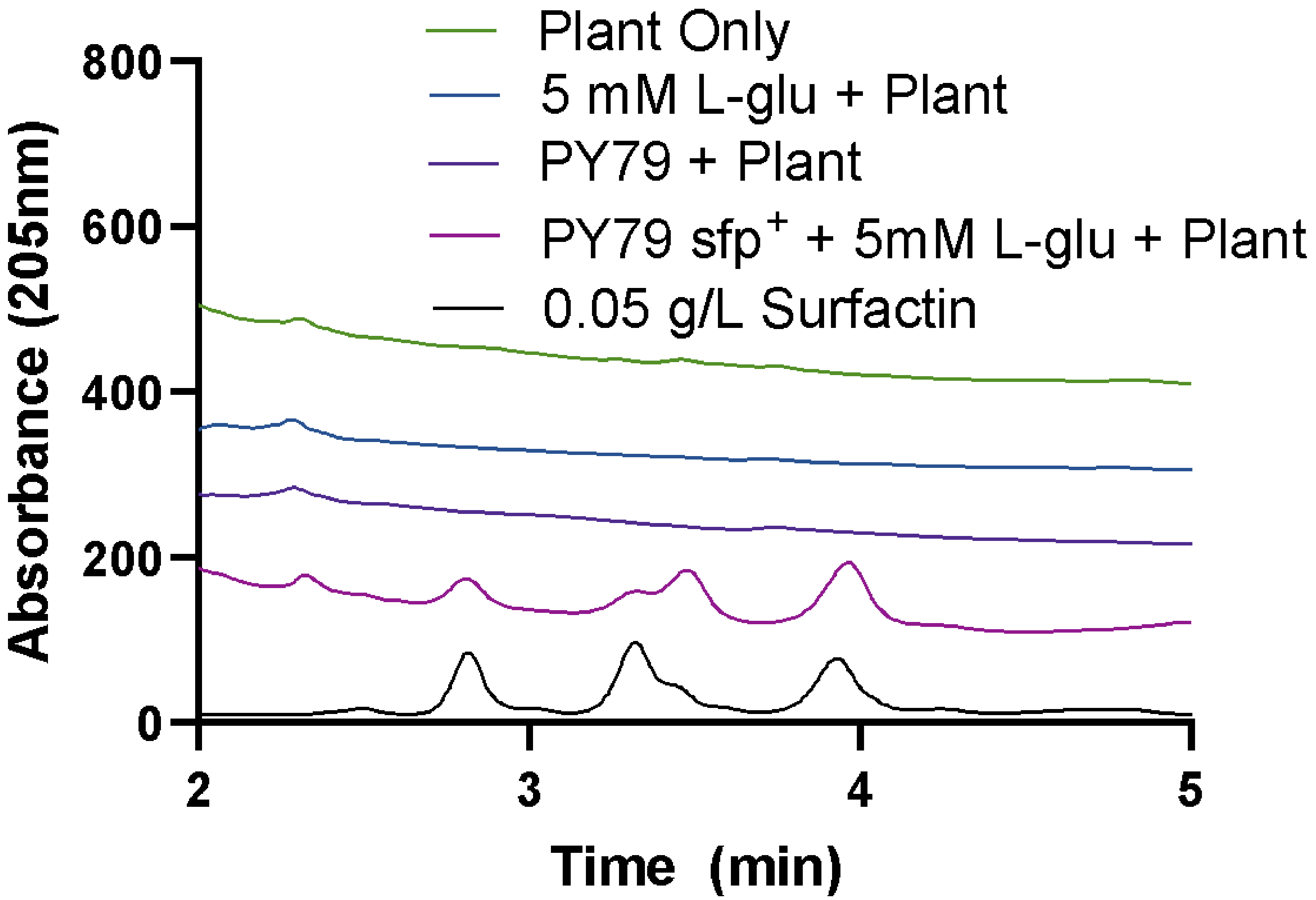

Plant Only
5 mM L-glu + Plant
PY79 + Plant
PY79 sfp+ + 5mM L-glu + Plant
0.05 g/L Surfactin
Absorbance (205nm)
800
600
400
200
0
2
3
4
5
Time (min)

SOM Figure 6

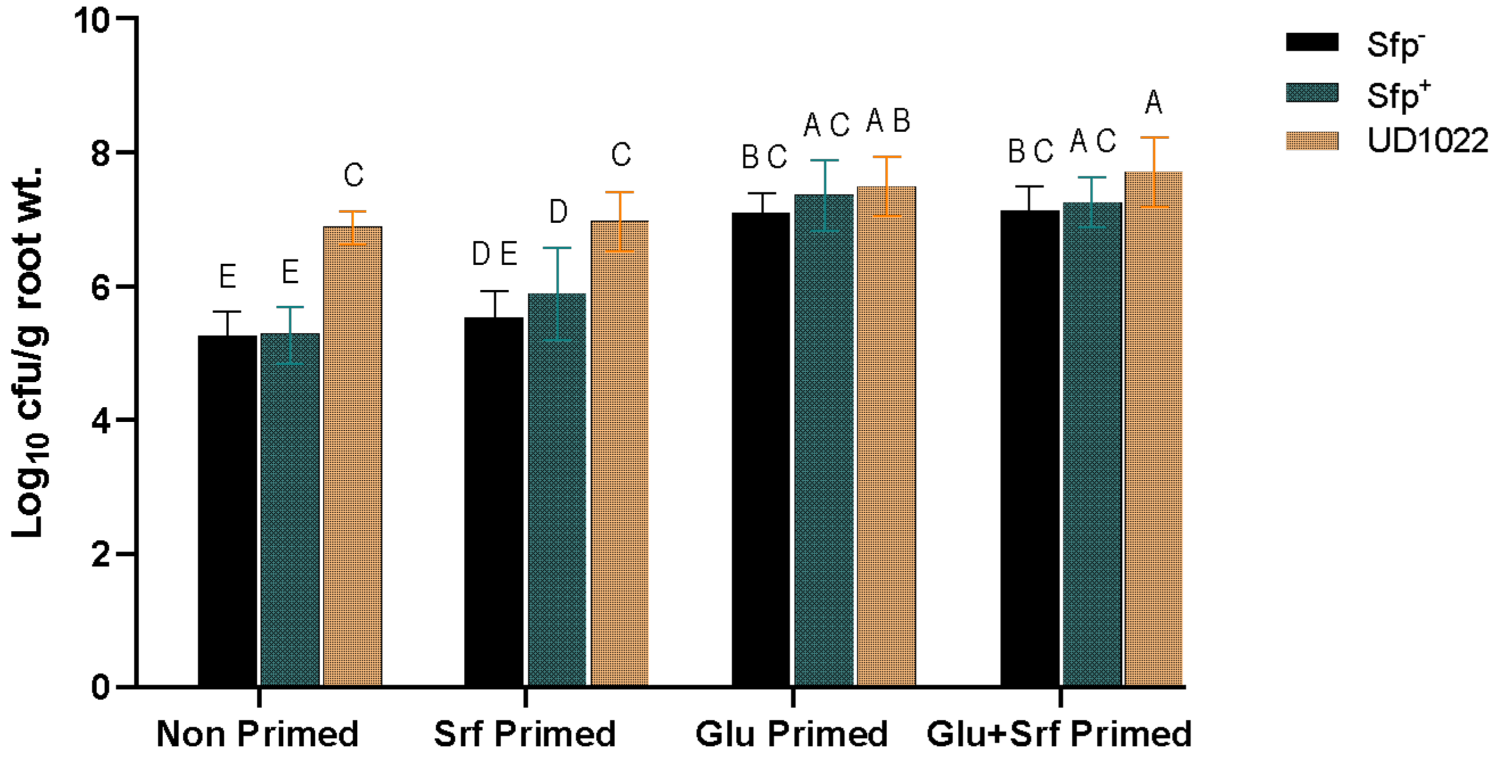

Sfp-
Sfp+
UD1022
E
E
C
D E
D
C
B C
A C
A B
B C
A C
A
Log_{10} cfu/g root wt.
10
8
6
4
2
0
Non Primed
Srf Primed
Glu Primed
Glu+Srf Primed

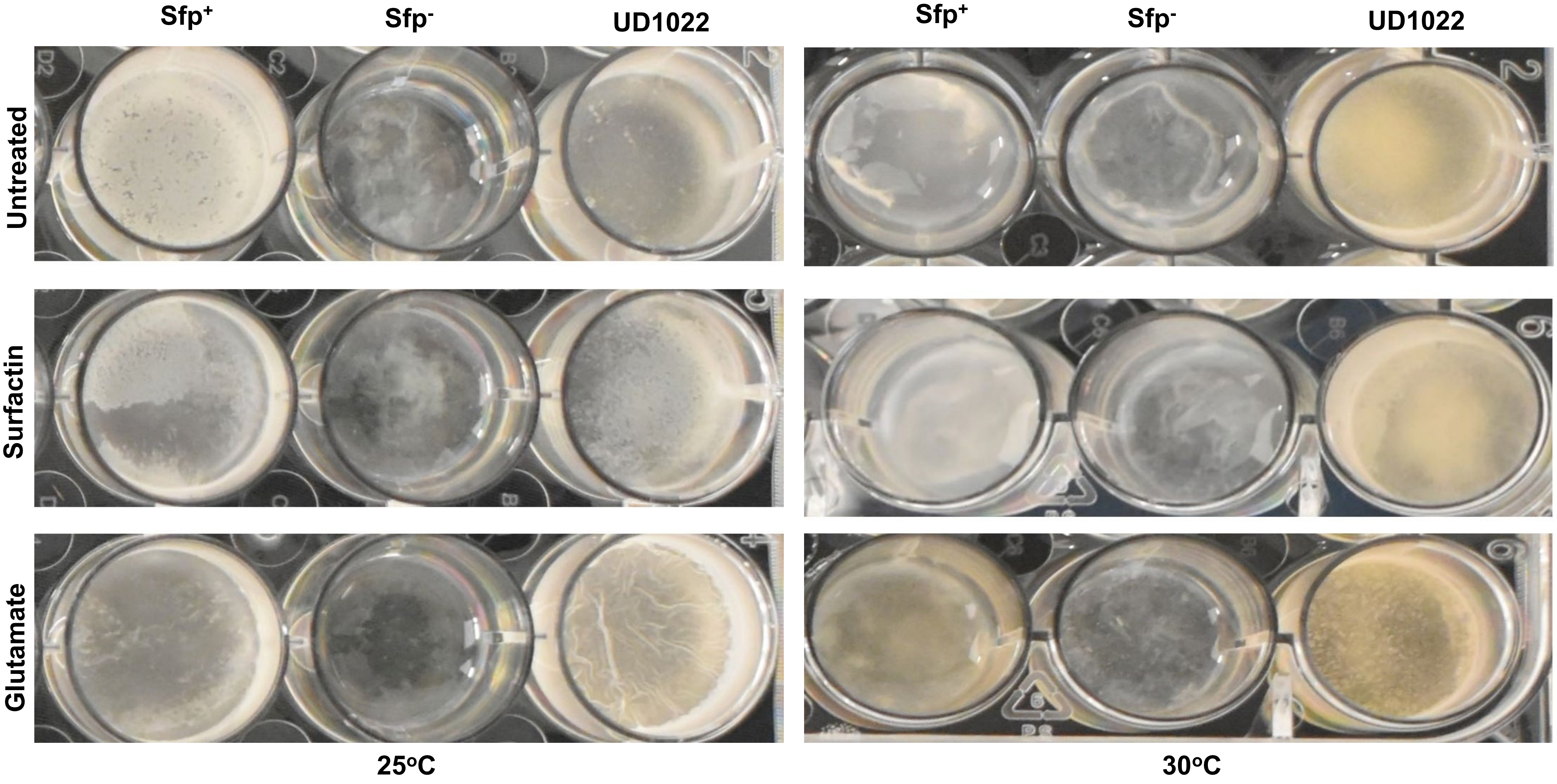
Sfp+
Sfp-
UD1022
Sfp+
Sfp-
UD1022
Untreated
Surfactin
Glutamate
25oC
30oC

SOM Figure 8

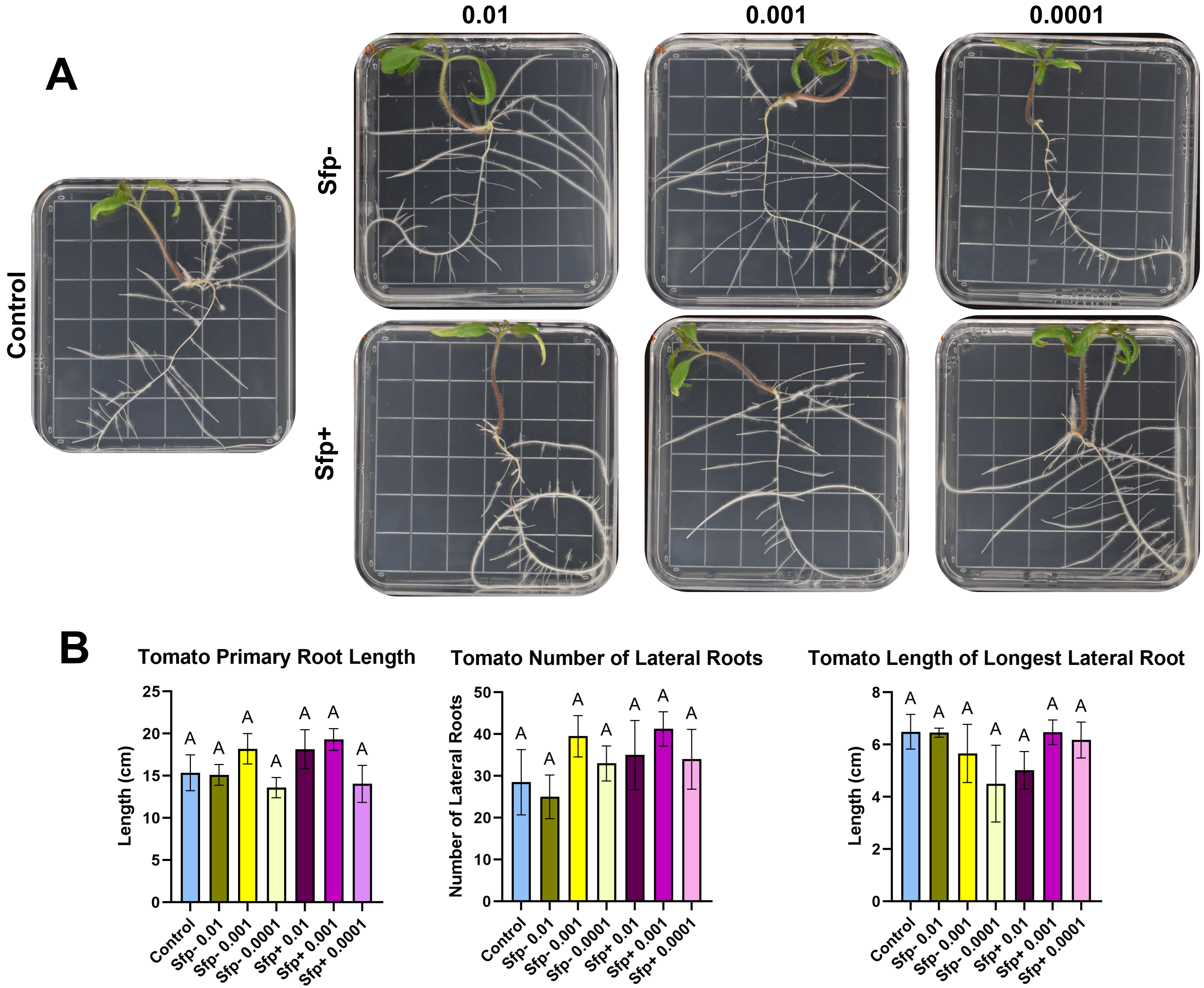
A
0.01
0.001
0.0001
Control
Sfp-
Sfp+
B
Tomato Primary Root Length
Length (cm)
Tomato Number of Lateral Roots
Number of Lateral Roots
Tomato Length of Longest Lateral Root
Length (cm)
Control
Sfp- 0.01
Sfp- 0.001
Sfp- 0.0001
Sfp+ 0.01
Sfp+ 0.001
Sfp+ 0.0001
A

SOM Figure 9

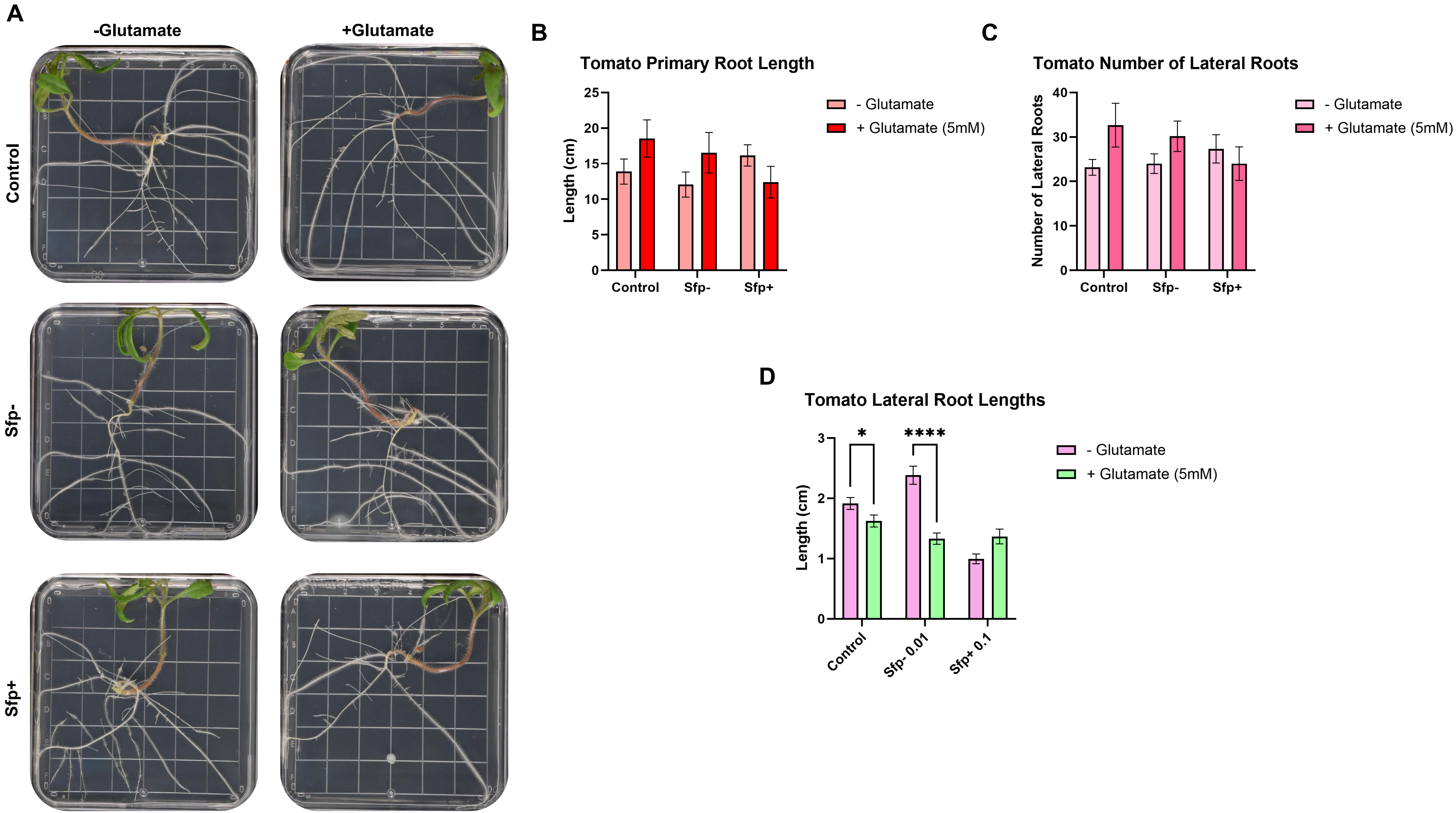

$OD_{600} = 0.01$

SOM Figure 10

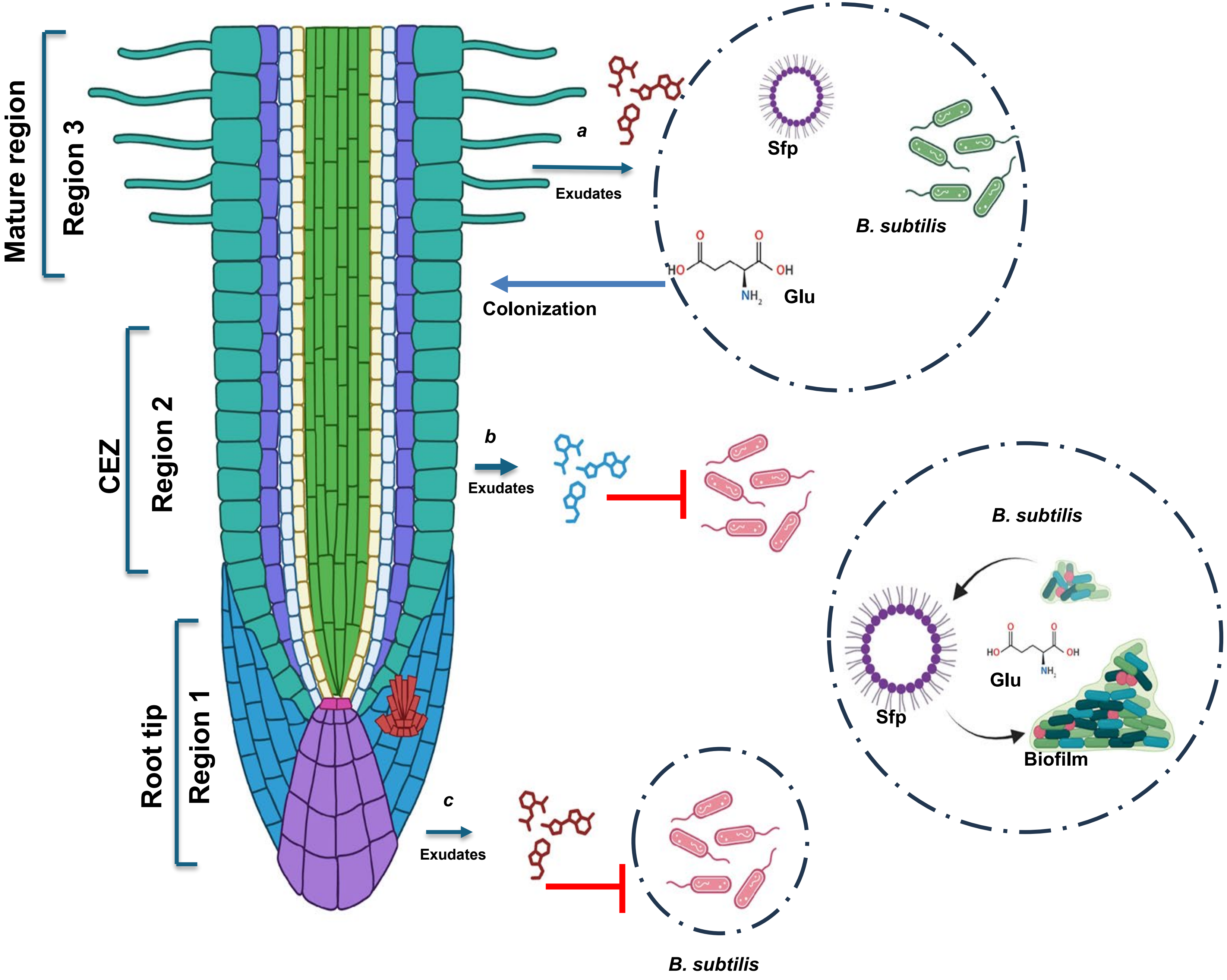

Supplement: Supplemental figures — Figures S1 to S10. [file spectrum.03181-25-s0002.pdf]
